# Circulating microRNA profiling is altered in the acute respiratory distress syndrome related to SARS-CoV-2 infection

**DOI:** 10.1038/s41598-022-10738-3

**Published:** 2022-04-28

**Authors:** Natalia Garcia-Giralt, Juan Du, Judith Marin-Corral, Marta Bódalo-Torruella, Fabiola Blasco-Hernando, Rosana Muñoz-Bermúdez, Miquel Clarós, Lara Nonell, Julia Perera-Bel, Marta Fernandez-González, Xavier Nogues, Luisa Sorli-Redó, Robert Güerri-Fernández

**Affiliations:** 1 Centro de Investigación Biomédica en Red de Fragilidad y Envejecimiento Saludable (CIBERFES), IMIM (Hospital del Mar Research Institute), Barcelona, Spain; 2 Critical Care Department, Hospital del Mar, Barcelona, Spain; 3 Critical Illness Research Group (GREPAC), IMIM, Barcelona, Spain; 4 Division of Pulmonary Diseases and Critical Care Medicine, University of Texas Health San Antonio, San Antonio, TX USA; 5 MARGenomics, IMIM (Hospital del Mar Research Institute), Barcelona, Spain; 6 Department of Infectious Diseases, Hospital del Mar-IMIM, Barcelona, Spain; 7 Internal Medicine Department, Hospital del Mar, Universitat Pompeu Fabra, Barcelona, Spain

**Keywords:** Infectious diseases, Epigenomics

## Abstract

One of the hallmarks of SARS-CoV-2 infection is an induced immune dysregulation, in some cases resulting in cytokine storm syndrome and acute respiratory distress syndrome (ARDS). Several physiological parameters are altered as a result of infection and cytokine storm. Among them, microRNAs (miRNAs) might reflect this poor condition since they play a significant role in immune cellular performance including inflammatory responses. Circulating miRNAs in patients who underwent ARDS and needed mechanical ventilation (MV+; n = 15) were analyzed by next generation sequencing in comparison with patients who had COVID-19 poor symptoms but without intensive care unit requirement (MV−; n = 13). A comprehensive in silico analysis by integration with public gene expression dataset and pathway enrichment was performed. Whole miRNA sequencing identified 170 differentially expressed miRNAs between patient groups. After the validation step by qPCR in an independent sample set (MV+  = 10 vs. MV− = 10), the miR-369-3p was found significantly decreased in MV+ patients (Fold change − 2.7). After integrating with gene expression results from COVID-19 patients, the most significant GO enriched pathways were acute inflammatory response, regulation of transmembrane receptor protein Ser/Thr, fat cell differentiation, and regulation of biomineralization and ossification. In conclusion, miR-369-3p was altered in patients with mechanical ventilation requirement in comparison with COVID-19 patients without this requirement. This miRNA is involved in inflammatory response which it can be considered as a prognosis factor for ARDS in COVID-19 patients.

## Introduction

Coronavirus disease 2019 (COVID-19) is a clinical syndrome caused by a RNA virus named as Severe Acute Respiratory Syndrome Corona Virus 2 (SARS-CoV-2). SARS-CoV-2 infection has a heterogenous disease course, ranging from asymptomatic or only mild symptoms to severe immunologic complications such as macrophage activation syndrome (MAS) resulting in cytokine storm syndrome and acute respiratory distress syndrome (ARDS). ARDS is the most severe form of acute lung injury. It is often induced as a result of sepsis or infectious injury (including SARS coronavirus, bird flu or human influenza viruses)^1^.

Ageing and underlying disorders (i.e., hypertension, chronic obstructive pulmonary disease, diabetes, obesity, cardiovascular disease) are clinical risk factors for developing ARDS, septic shock, coagulation dysfunction and death as a response to viral infections such as SARS-CoV-2^2^.

In normal conditions, virus-infected cells are destroyed by NK cells of the innate immunity and cytolytic CD8^+^ T-cells of the adaptive immunity. This induces antigen presenting cells and cytotoxic T-cells apoptosis to avoid unnecessary activation once the antigenic activity is over. However, if a defect occurs in the lymphocyte cytolytic activity, likely due to genetic factors and other acquired conditions, this may lead to the inability of NK and cytolytic CD8^+^ T-cells to lyse infected and activated antigen-presenting cells. This may result in prolonged and exaggerated interactions between innate and adaptive immune cells with secretion of many pro-inflammatory cytokines, including Tumor Necrosis Factor (TNF), interferon-γ, interleukin (IL)-1, IL-6, IL-18, and IL-33 in an uncontrolled manner causing a cytokine storm, ARDS, and multiorgan failure^3^.

One of the hallmarks of SARS-CoV-2 infection is an induced immune dysregulation. Severe lymphocytopenia is a very early sign of the disease, preceding lung involvement, which tends to normalize as the patient improves^4^. In parallel, monocytes and macrophages are increased, contributing to raised levels of proinflammatory cytokines such as IL-6, IL-1, TNF-α, and IL-8, which in some patients turn out to be a cytokine storm. The great majority of the inflammatory cells infiltrating the lungs are monocytes and macrophages^5^.

There are many potential factors regulating this immune response. Among them, microRNAs (miRNAs) are small non-coding RNA molecules that are partially complementary to messenger RNA and downregulate gene expression in a variety of manners^6^. Interestingly, miRNA expression is modified by different deleterious processes such as viral infection^7^ and several immune system alterations^8^.

In this context, patients who require invasive mechanical ventilation display a general deterioration involving organic failure. A number of physiological parameters are altered as a result of infection and cytokine storm. Among them, miRNA levels might reflect this poor condition since they play a significant role in immune cellular performance including inflammatory responses^9–11^. Few studies attempted to identify microRNAs involved in the ARDS related to COVID-19. Among them, a study performed by Tang et al.^11^ found several miRNAs specific for severe COVID‐19 which may serve as potential biomarkers and therapeutic targets such as miR‐15b‐5p. Identification of dysregulated miRNAs during ARDS may help to understand molecular mechanisms and immunity pathways involved in regulation of excessive inflammatory responses and in the pathogenesis of many human inflammatory diseases to develop potentially useful treatments.

## Methods

### Patients

A case–control study was conducted with COVID-19 patients admitted in the Hospital del Mar, Barcelona, Spain. All participants (n = 48) were hospitalized in COVID wards due to severe COVID-19 symptoms and a subset were admitted to Intense Care Unit (ICU) according to criteria listed in supplemental material. Of them, 28 patients were included in the discovery sample and analysed by next generation sequencing (NGS): Fifteen patients required mechanical ventilatory suport (MV+) and they were admitted to ICU whereas the other thirteen patients remained in COVID ward without mechanical ventilation (MV−). Co-morbidities and cumulative risk factors of each individual patients was reported in Supplementary Table 1. Twenty patients were included in the validation step by qPCR: 10 MV+ and 10 MV−.

All hospitalized patients received the hospital’s standard-of-care therapy at the time of the study (first wave of COVID-19 pandemic in Spain), consisting in hydroxychloroquine 400 mg/24 h first day and 200 mg/24 h 4 days with azithromycin 500 mg/24 h 3 days, plus ceftriaxone 1 or 2 g/24 h 7 days when there was bacterial superinfection. Patients with severe or critical conditions of pulmonary affectation or suspected cytokine storm were additionally treated with dexamethasone bolus (20 mg/day × 4 days) according to hospital protocol. Demographic and anthropometric variables, full medical history and blood samples were obtained for all participants at time of hospital admission. The blood draw was done systematically in fasting between 8 and 10am. A tube BD Vacutainer® Plus Serum Tubes / CAT Tubes that are coated with silicone and micronized silica particles to accelerate clotting were used. We followed the manufacturer instructions for sample preparation and processing in all the samples. In addition, the same personnel obtained the samples and processed them to minimize interindividual variability.

Statistical analysis of clinical variables was done using SPSS23. Welch Two Sample t-test and Fisher's Exact Test were used to make pairwise comparisons between groups of quantitative and qualitative variables respectively. The sum risk factors, which encompasses all comorbidities described in Table 1, was analysed by Wilcoxon rank sum test. Results with *p* < 0.05 were considered statistically significant.

**Table 1:** Patient characteristics stratified according to mechanical ventilation (MV) requirement.

MV requirement	Yes (N = 15)	No (N = 13)	*p* value
**Discovery sample (NGS analysis)**			
Mean age (years) ± SD	49 ± 8.7	44 ± 12	0.231
Gender (% male)	9 (60%)	7 (53.8%)	0.387
Dead	1	0	0.478
HBP (n(%))	9 (60%)	1 (7.7%)	0.011
CAD (n(%))	1 (6.7%)	0 (0.0%)	0.212
Obesity (n(%))	7 (46.7%)	0 (0.0%)	0.011
Cancer* (n(%))	0 (0.0%)	2 (15.4%)	0.21
Chronic kidney disease (n(%))	3 (20%)	0 (0.0%)	0.091
Chronic infections (n(%))	0 (0.0%)	1 (7.7%)	0.214
Chronic respiratory diseases (n(%))	5 (33.3%)	2 (15.4%)	0.109
Diabetes Mellitus (n(%))	4 (26.7%)	0 (0.0%)	0.091
Sum risk factors (Mean ± SD)	2.6	1.2 ± 1.5	0.041
IL-6 (pg/ml) (Mean ± SD)	798 ± 699	52.5 ± 33.1	0.002

MV requirement	Yes (N = 10)	No (N = 10)	*p* value
**Validation sample (qPCR analysis)**			
Mean age (years) ± SD	63 ± 9	55 ± 12	0.131
Gender (% male)	5 (50%)	6 (60%)	0.361
Dead	3 [30]	0	0.060
HBP (n(%))	2 (20%)	5 (50%)	0.160
CAD (n(%))	1(10%)	1 (10%)	1
Obesity (n(%))	3 (30%)	5 (50%)	0.361
Cancer* (n(%))	0	0	1
Chronic kidney disease (n(%))	1 (10%)	0	0.305
Chronic infections (n(%))	0	1	0.350
Chronic respiratory diseases (n(%))	4 (40%)	2(20%)	0.260
Diabetes Mellitus (n(%))	4 (40%)	3 (30%)	0.630
Sum risk factors (Mean ± SD)	3.1 ± 2.1	2.3 ± 1.4	0.106
IL-6 (pg/ml) (Mean ± SD)	967 ± 933	37.5 ± 44	0.001

*SD* standard deviation, *HBP* high blood pressure, *CAD* cardiovascular disease, *NS* non-significant.

*Previous cancer or current.

The study protocol was approved by the ethics committee of Parc de Salut Mar (CEIm 2020/9232/I) and it was carried out in accordance with the Declaration of Helsinki and the Good Clinical Practice guidelines of the International Conference on Harmonization. All patients were verbally informed about the treatments, by formally obtaining their consent, and its acceptance was recorded in the electronic medical record of the Hospital.

## Discovery stage

### Next generation sequencing (NGS) of serum microRNAs

#### miRNA isolation, library construction and sequencing

RNA was extracted from serum samples using miRNeasy Serum/Plasma kit (QIAGEN). MiRNA quantity and purity were determined on Qubit miRNA assay and RNA integrity was assessed using Agilent 2100 Bioanalyzer (Agilent Technologies). Small RNA libraries were prepared by processing 16 and 14 samples per batch (conditions well distributed) using QIAseq miRNA Library Kit (QIAGEN) according to the manual QIAseq miRNA Library Kit Handbook for Illumina® (QIAGEN, Version 1.0, 12/18). The suggested parameters for serum/plasma samples were: 5 μl of initial sample, different dilutions of adaptors/primers and 22 PCR cycles. Quality control of the libraries was done using Bioanalyzer DNA High Sensitivity and Qubit DNA High Sensitivity, 28 of 29 libraries passed the quality control and subsequently were sequenced. Library quantifications were performed with KAPA Library Quantification kit on ABI Fast 7500 RT-PCR instrument. All libraries were pooled and sequenced on the Illumina NextSeq 500 platform for single read 75 cycles with an expected coverage of 14 M per sample.

#### Data analysis

Qiagen adapters were removed (allowing 1 mismatch) and UMI sequences were retained with UMI tools version 0.5.4 (extract module). Reads shorter than 15 bp were removed with Cutadapt version 2.1.

Reads in the fastq files were mapped with STAR version 2.6.0^12^ against miRBase v22.1. The table of counts was obtained with featureCounts function in the package Subread, version 1.6.4^13^ and posterior counting of unique UMIs with UMI tools version 0.5.4 (count, default method “directional”). The differential gene expression analysis (DEG) was assessed with voom + limma in the limma package version 3.44.1^14^ and using R version 4.0.0.

Genes having less than 3 counts in at least 2 samples were excluded from the analysis. Raw library size differences between samples were treated with the weighted “trimmed mean method” TMM^15^ implemented in the edgeR package^16^. The normalized counts were used in order to make unsupervised analysis, PCA, clusters and heatmaps. For the differential expression (DE) analysis, read counts were converted to log2-counts-per-million (logCPM) and the mean–variance relationship was modelled with precision weights using voom approach in limma package using a linear model design adjusting for the sum of risk factors. The AUC (Area Under The Curve)-ROC (Receiver Operating Characteristics) curve for miR-369-3p was obtained using SPSS 22 and STATA software.

## Validation stage

### qPCR analysis of candidate miRNAs

All experiments were conducted by QIAGEN Genomic Services.

#### Sample preparation

An aliquot of 200 μl of serum was transferred to a FluidX tube and 60 μl of Buffer RPL containing 1 μg carrier-RNA and RNA spike-in template mixture were added to the sample and mixed for 1 min and incubated for 7 min at room temperature, followed by addition of 20μL Buffer RPP. Total RNA was extracted from the samples using miRNeasy Serum/Plasma Advanced Kit; high-throughput bead based protocol v.1 (Hilden, Germany) in an automated 96 well format. The purified total RNA was eluted in a final volume of 50 μl. miRNA real-time qPCR 2 μl RNA was reverse transcribed in 10 μl reactions using the miRCURY LNA RT Kit (QIAGEN).

cDNA was diluted 50× and assayed in 10 μl PCR reactions according to the protocol for miRCURY LNA miRNA PCR; each miRNA was assayed once by qPCR on the miRNA Ready-to-Use PCR, Custom panel using miRCURY LNA SYBR Green master mix. Negative controls excluding template from the reverse transcription reaction was performed and profiled like the samples. The amplification was performed in a LightCycler® 480 Real-Time PCR System (Roche) in 384 well plates. The amplification curves were analyzed using the Roche LC software, both for determination of Cq (by the 2nd derivative method) and for melting curve analysis.

Hemolysis was tested in all samples using miR-451 (is expressed in red blood cells) and miR-23a-3p (relatively stable in serum and plasma and not affected by hemolysis). dCq(23a-451) was lower than 7 in all samples showing minimal signs of hemolysis.

#### Data analysis

The amplification efficiency was calculated using algorithms similar to the LinReg software. All assays were inspected for distinct melting curves and the Tm was checked to be within known specifications for the assay. Furthermore, assays must be detected with 5 Cq less than the negative control, and with Cq < 37 to be included in the data analysis. All data was normalized with hsa-miR-502-3p and hsa-miR-23a-3p using the average of the assays detected in all samples (n = 20 samples).

### Pathway enrichment analysis

In silico analysis of predicted pathways for the miR-369-3p was performed using miTALOS 2.0 (http://mips.helmholtz-muenchen.de/mitalos/#/search), miRSystem (http://mirsystem.cgm.ntu.edu.tw/index.php), and mirPath v.3 (http://snf-515788.vm.okeanos.grnet.gr/#mirnas=hsa-miR-369-3p&methods=microT-CDS&selection=0).

### Integration with public gene expression dataset

Overmyer et al.^17^ performed RNA-Seq from leukocytes from 102 patients with COVID-19 (51 patients needed for admission into ICU and 51 hospitalized in COVID-19 floor). We downloaded the count matrix from GEO (GSE157103) and filtered genes having less than 10 counts in at least 50 samples. Read counts were converted to log2-counts-per-million (logCPM) and the mean–variance relationship was modelled with precision weights using voom approach in limma package adjusting by sex and age (as done in the original publication). The contrast performed for this analysis was ICU.requirement vs. no ICU.

Genes were considered differentially expressed (DE) if the adjusted *p* value < 0.05 and |FC| > 1.5. To find which DE genes could potentially be regulated by the miR-369-3p, we assumed that down-regulation in miR-369-3p will lead to up-regulation in genes. Hence, we retrieved targets for miR-369-3p from miRTarBase (v.8), miRDB (v.6) and TargetScan (v.7.2) and intersected these targets with DE genes (up-regulated in ICU patients).

Finally, we performed enrichment analysis of the list of intersected genes implemented in clusterProfiler^18^ R package version 3.18.0 using the molecular signatures database collection c5.bp^19,20^: Gene sets derived from the Biological Process Gene Ontology (GO), version 7.2. Gene sets were considered significantly enriched if the adjusted *p* value < 0.05 (after multitesting correction^21^).

## Results

### Sample description

The anthropometric features of the MV+ and MV− groups are shown in Table 1. Statistical differences in high blood pressure and obesity were observed between both groups. In addition, an increased inflammatory component was detected in MV+ group according to IL-6 levels measured a baseline. Otherwise, there were not significant differences between groups at time of hospital admission in the different cell counts, which included: Leukocytes, Neutrophils, Lymphocytes, Eosinophils, Hemoglobin, Hematocrit, CD4 and CD8 cells.

### NGS analysis of circulating miRNAs

MicroRNA levels were analyzed in serum samples using next generation sequencing in the discovery stage (GSE182183). Overall, the mean of normalized counts of detected miRNAs ranges from 17 to − 1.64 where the top ten more expressed miRNAs were miR-16-5p, let-7b-5p, miR-486-3p, miR-486-5p, let-7a-5p, let-7i-5p, miR-126-3p, let-7f-5p, miR-223-3p, and miR-142-3p (all of them over 14 counts). The ten most rare miRNAs were hsa-miR-1266-3p, hsa-miR-3940-5p, hsa-miR-410-5p, hsa-miR-5001-5p, hsa-miR-6507-5p, hsa-miR-381-5p, hsa-miR-34c-3p, hsa-miR-770-5p, hsa-miR-1193, and hsa-miR-6796-3p. Whole microRNA expression was compared between COVID-19 patients with mechanical ventilation requirements versus COVID-19 patients without this requirement. A total of 170 miRNAs were differentially expressed between MV+ and MV− patients with a value of log-fold change larger than 1 and *p* value below 0.05 (Supplementary Table 2). The most abundant miRNAs were hsa-miR-142-3p, hsa-miR-122-5p, hsa-miR-26b-5p while hsa-miR-937-5p, hsa-miR-6796-5p, hsa-miR-34c-3p were the least detected miRNAs (Supplemental Table 2). Interestingly, the miR-142-3p is involved in the post-transcriptional regulation of IL-6 expression which mainly has functions in inflammatory processes, and it shows an abnormal production in patients with COVID-19^22^. The miR-122-5p is elevated in liver injury^23^ and coronary heart disease^24^ and it is upregulated in MV+ patients (Supplemental Table 2). The miR-26b-5p is involved in adipocyte differentiation and could have pathophysiological roles in obesity and its related metabolic diseases^25^. On the other hand, among the most significant differentially expressed miRNAs, miR-34c-3p is also the least represented miRNA in serum samples, especially in patients with MV−. This miRNA has been described as likely involved in pulmonary fibrosis^26^. However, these low levels of expression could lead to significant spurious associations, and therefore differences found between groups should be taken with caution.

The top ten upregulated and top ten downregulated miRNAs in MV+ are shown in Table 2. Corresponding heatmap is shown in Supplementary Figure 1.

**Table 2:** The top ten upregulated and top ten downregulated miRNAs in MV+ patients compared to MV− patients in the discovery sample using NGS.

	Chr	Start	End	logFC MV+ vs MV−	*p* value MV+ vs MV−
**Downregulated miRNAs**					
hsa-miR-144-3p	chr17	28,861,548	28,861,567	− 1.952	0.011
hsa-miR-15a-3p	chr13	50,049,130	50,049,151	− 1.941	0.003
hsa-miR-374a-3p	chrX	74,287,295	74,287,316	− 1.886	0.002
hsa-miR-181c-5p	chr19	13,874,725	13,874,746	− 1.849	0.001
hsa-miR-374a-5p	chrX	74,287,325	74,287,346	− 1.831	0.004
hsa-miR-1277-5p	chrX	118,386,401	118,386,424	− 1.795	0.004
hsa-miR-369-3p	chr14	101,065,641	101,065,661	− 1.783	0.005
hsa-miR-146a-3p	chr5	160,485,408	160,485,429	− 1.761	0.011
hsa-miR-556-3p	chr1	162,342,600	162,342,621	− 1.746	0.007
hsa-miR-16-1-3p	chr13	50,048,985	50,049,006	− 1.716	0.009
**Upregulated miRNAs**					
hsa-miR-193b-5p	chr16	14,303,980	14,304,001	2.650	0.001
hsa-miR-4669	chr9	134,379,449	134,379,470	2.512	0.009
hsa-miR-193a-5p	chr17	31,560,016	31,560,037	2.168	0.0001
hsa-miR-4697-3p	chr11	133,898,506	133,898,529	1.957	0.048
hsa-miR-4516	chr16	2,133,120	2,133,136	1.955	0.002
hsa-miR-1228-3p	chr12	57,194,555	57,194,574	1.932	0.007
hsa-miR-3182	chr16	83,508,349	83,508,365	1.862	0.0005
hsa-miR-150-3p	chr19	49,500,797	49,500,818	1.826	0.005
hsa-miR-885-3p	chr3	10,394,499	10,394,520	1.753	0.019
hsa-miR-34c-3p	chr11	111,513,484	111,513,505	1.690	0.018

miRNA name: Annotations related to the gene (miRNA) according to miRBase v22.1, genome version GRCh38. *p* value obtained with the moderated t-test.

*Chr* chromosome, *logFC* log2 fold change, *MV* mechanical ventilation requirement.

### qPCR analysis of candidate miRNAs

Twenty miRNAs underwent validation in a different set of samples (10 MV+ and 10 MV−) by qPCR: hsa-miR-374a-5p, hsa-mir-374a-3p, hsa-mir-144-3p, hsa-mir-15a-3p, hsa-mir-556-3p, hsa-mir-150-3p, hsa-mir-181c-5p, hsa-mir-16-1-3p, hsa-mir-1277-5p, hsa-mir-885-3p, hsa-mir-146a-3p, hsa-mir-34c-3p, hsa-mir-369-3p, hsa-mir-4516, hsa-mir-193a-5p, hsa-mir-125a-3p, hsa-mir-193b-5p, hsa-mir-4488, hsa-mir-320a, and hsa-mir-206.

MiRNAs were selected according to the following criteria: differentially expressed in the discovery step, available Qiagen probes, and previously associated with an infectious or inflammatory condition (according to available literature^9,11^).

**Figure 1:**
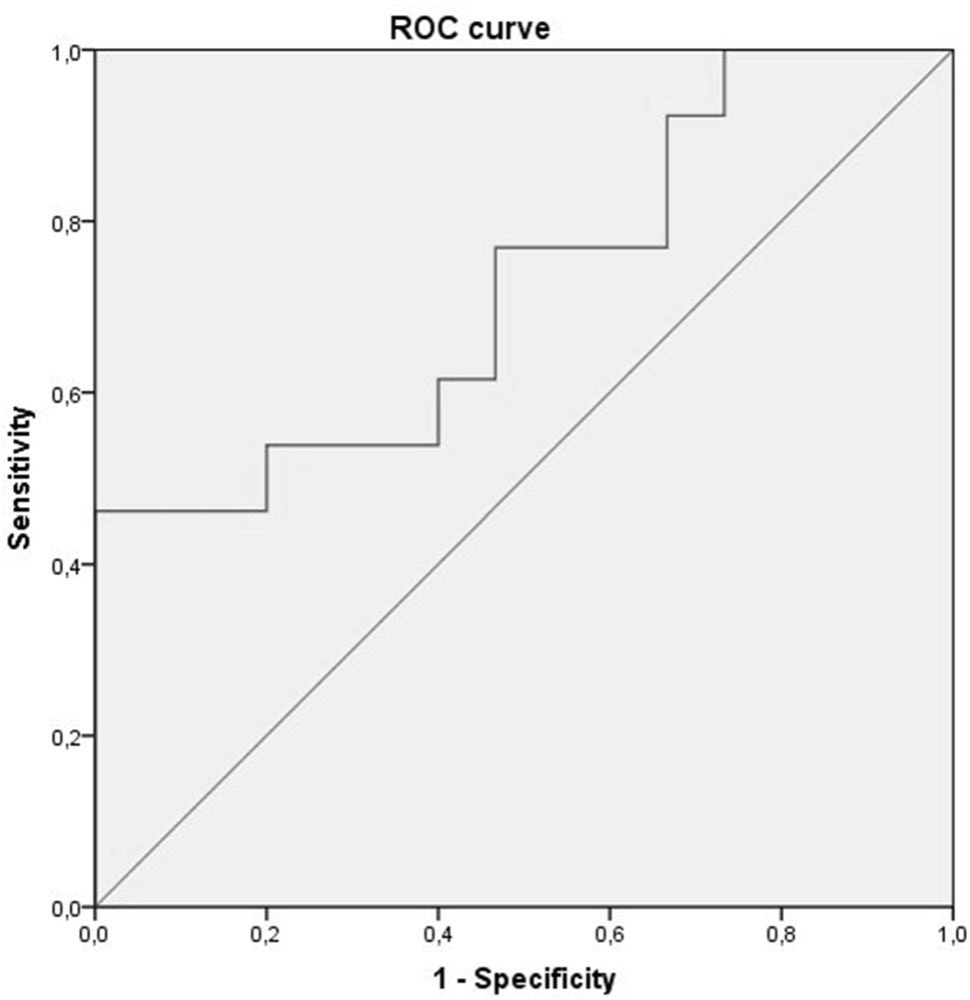
ROC curve for the miR-369-3p. Expression levels were quantified by NGS.

Finally, the miR-369-3p was validated (Supplementary Table 3): it was found under-expressed in MV+ patients (Fold change − 2.7; *p* value = 0.0061). ROC curves and AUCs were used to assess the discriminative accuracy of the miR-369-3p (Fig. 1). The AUC (95% CI) for discriminating MV− vs. MV+ patients was 0.72 (0.53–0.91), standard error = 0.098, and *p* value = 0.045. Using a cut-off point of 2.75 in miR-369-3p levels according to NGS results, the sensitivity was 77% with a specificity of 53.3%.

### Pathway enrichment analysis

**Figure 2:**
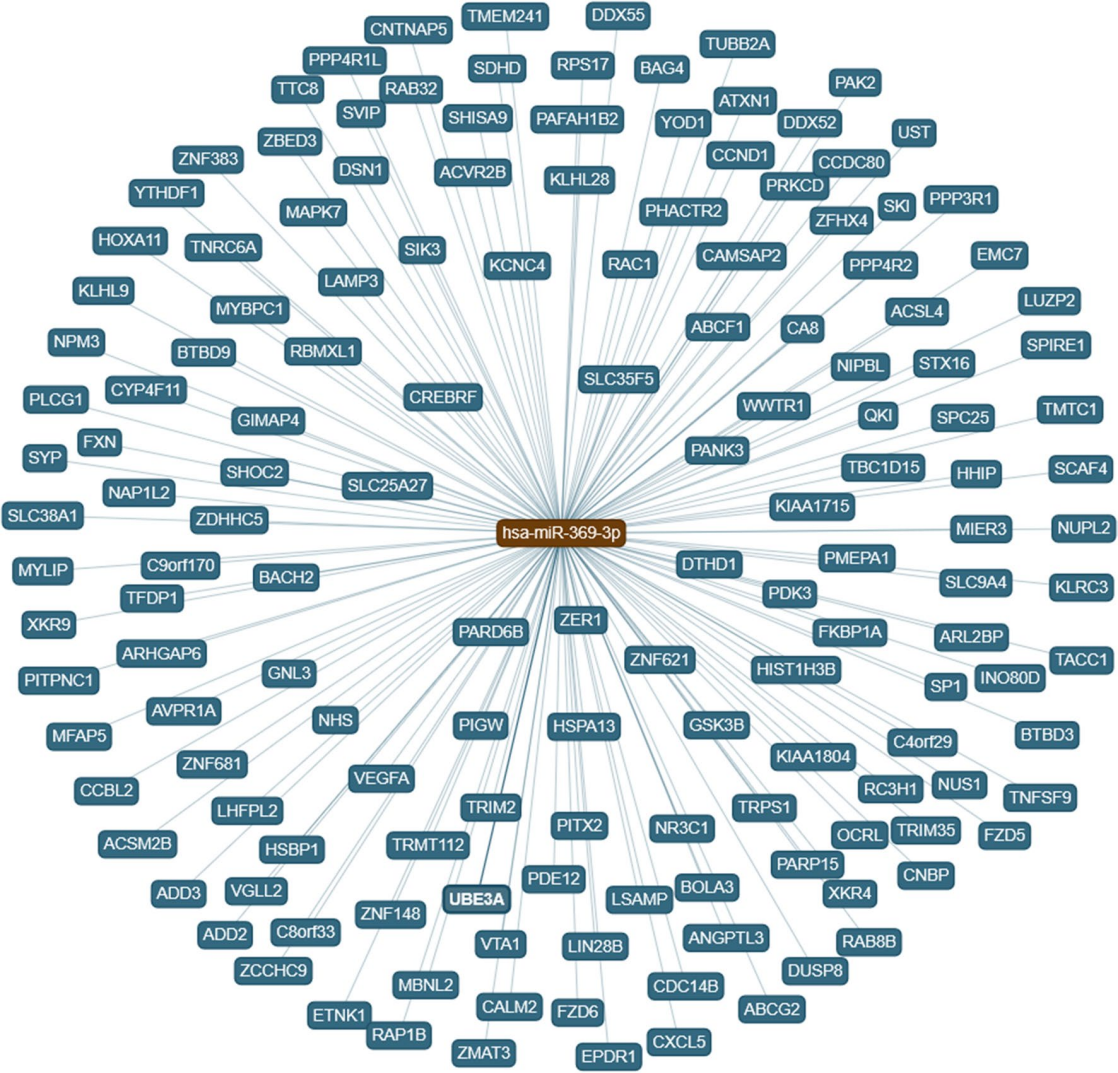
Predicted targets for miR-369-3p using *Chair for Bioinformatics* at the University of Saarland. https://ccb-web.cs.uni-saarland.de/mirtargetlink/network.php?type=miRNA&qval=hsa-miR-369-3p.

**Figure 3:**
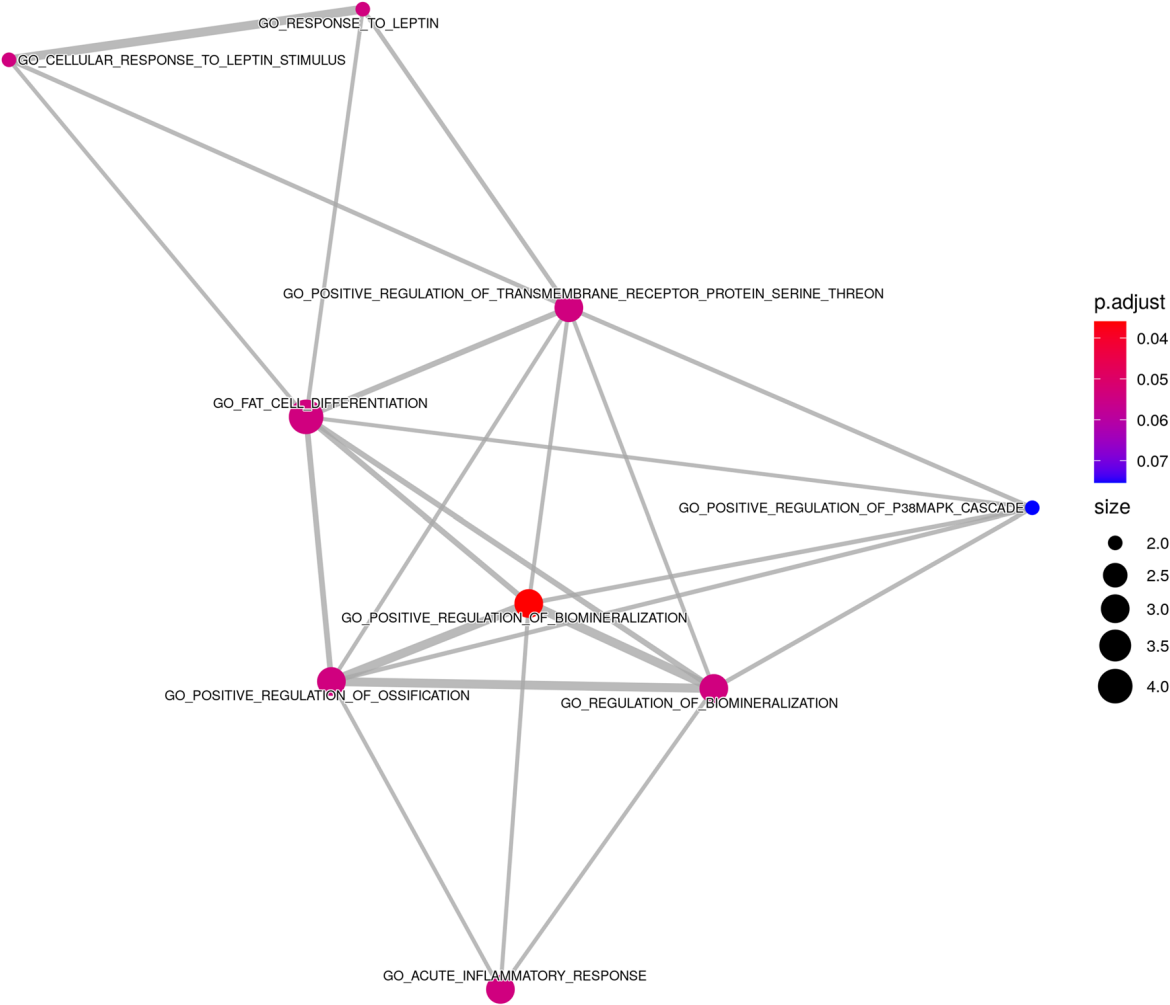
Pathway enrichment analysis of miR-369-3p target genes that were found significantly up-regulated in MV+ patients compared to MV− patients in the public gene expression dataset GSE157103. The functional enrichment analysis was performed using the R package, clusterProfiler. The figure shows the enrichment map of the inter-relation between enriched pathways.

Pathway enriched results for the miR-369-3p displayed diverse significant signaling pathways, some of them related to immune system: T-Cell Receptor and Co-stimulatory Signaling (*p* value = 0.002), IL-4 Signaling Pathway (*p* value = 0.008), Notch signaling pathway (*p* value = 0.035), and TGF-beta signaling pathway (*p* value = 0.003). Figure 2 shows predicted targets for the miR-369-3p using Chair for Bioinformatics at the University of Saarland^27^. MirPath v.3 analysis, considering TarBase v7.0 that provides high-quality manually curated experimentally validated miRNA:gene interactions, the most significant pathway was circadian entrainment (*p* value < 0.001) but the Hedgehog signaling pathway was also significant (*p* value = 0.016). After integrating with gene expression results from GSE157103 (Supplementary Table 4), the most significant GO enriched pathways were fat cell differentiation, regulation of transmembrane receptor protein Ser/Thr, regulation of biomineralization and ossification, and acute inflammatory response (Fig. 3).

## Discussion

The aim of this study was to identify microRNAs altered in ARDS in the context of SARS-CoV-2 infection. Circulating miRNAs in patients who underwent ARDS and needed mechanical ventilation were analyzed in comparison with patients who had COVID-19 but without ICU requirement. First of all, NGS results identified 170 miRNAs differentially expressed between patient groups in a discovery sample. Twenty miRNAs with the best hit scores were chosen for validation by qPCR in an independent set of samples. Finally, the miR-369-3p was found significantly reduced in patients with ARDS. In silico analysis of this miRNA predicted a putative role in the regulation of immune pathways for example by targeting genes in the T-Cell Receptor and Co-stimulatory signaling, IL-4 signaling, Notch signaling, and TGF-beta signaling pathways. Considering only validated targets, it is interesting to highlight the Hedgehog signaling pathway that is involved in a wide variety of functions and in some diseases like pulmonary fibrosis^28^. Moreover, differentially expressed genes between ICU requirement vs. no ICU patients (GSE157103) that are potentially regulated by the miR-369-3p are enriched in pathways involved in the immune system like Serine/threonine kinase receptors which are mediators of TGF-beta family signals^29^, and in the acute inflammatory response.

Previous literature validated the miR-369-3p in its role in the immune system. For example, miR-369-3p was found upregulated in severe and/or recurrent Herpes Simplex Virus infection^30^. In this study, miR-369-3p inhibition in NK-92 cells resulted in profound upregulation of 4 genes (APOBEC3G, MAP2K3, MAVS and TLR7) and downregulation of 36 genes taking part in antiviral response or associated with signaling pathways of Toll-like receptors (TLR), NOD-like receptors, the retinoic acid-inducible gene I (RIG-I)-like receptors (RLRs) and type I IFN-related response^30^.

Accordingly, miR-369 inhibition significantly increased cell apoptosis and inflammatory factor production triggered by hypoxia by targeting TRPV3^31^.

Another study demonstrated that the upregulation of miR-369-3p suppresses the LPS-induced inflammatory response, reducing C/EBP-β, TNFα and IL-6 production. Moreover, it was suggested that miR-369-3p plays a key role in negatively regulating the LPS-induced Dendritic cells responses mainly targeting iNOS. They found that the upregulation of miR-369-3p decreased the release of TNFα, IL-6, IL-12, IL-1α, IL-1β in response to LPS, and increased the production of anti-inflammatory cytokines such as IL-10 and IL-1RA^32^. Furthermore, a downregulation of miR-369-3p has been previously reported in Crohn’s disease plasma compared with control plasma^33^. It is noteworthy that all these findings are in line with our results where miR-369-3p is significantly decreased in patients with poorer COVID-19 prognosis suggesting this microRNA as a potential central player in the inflammatory response. Accordingly, the MV+ group had significantly higher IL-6 making this hypothesis more plausible and showing a potential association between these facts. Interestingly, a recent study found that miR-369-3p expression was decreased in the lung tissues of mice with induced idiopathic pulmonary fibrosis. By silencing the lncRNA DLEU2, the idiopathic pulmonary fibrosis was suppressed through upregulating miR-369-3p expression and downregulating TRIM2 expression^34^.

On the other hand, the miR-369-3p has a target site in the SARS-CoV-2 genome^35^ that could suggest an antiviral activity of this miRNA. Hence, a dual action of this miRNA, both on immune system and viral performance could be involved in the COVID-19 severity. Interestingly, the miR-369-3p does not target other SARS viruses even though they share approximately 78.7% of the genome^35^.

As a limitation, we cannot know if the under-expression of miR-369-3p in MV+ patients is cause or consequence of the condition, since we only analyzed the microRNA signature at a single time point, therefore we only have a vision of this particular moment. Noteworthy, samples were obtained at hospital admission, prior to ARDS and ICU requirement. Hence, our study points out that miR-369-3p is altered in severe COVID-19 patients prior developing ARDS and therefore it can play a role in this context. Thus, in an intra-hospital setting, miR-369-3p could be used as a potential prognostic marker in a time window between hospital admission and ICU requirement. Further studies should be addressed to elucidate whether miR-369-3p was downregulated prior to cytokine storm and therefore whether miR-369-3p can predict the transition from a severe to a critical phase of the disease.

Moreover, since COVID-19 severity is highly associated with other comorbidities, as for example obesity and high blood pressure, we cannot rule out that miR-369-3p levels were also associated with a multiple comorbidity/risk factors state, instead specifically associated with COVID-19-related ARDS. However, results from discovery sample were adjusted for the sum of risk factors in an attempt to minimize the effect of co-morbidities and a validation sample was chosen with minimum differences between groups, or at least, with no statistical differences in the most known COVID-19-related comorbidities.

These results and previous studies collectively contribute to a better knowledge of miR-369-3p and their target pathways which may be considered a potential target for new molecular therapeutic approaches^36,37^. Importantly, further studies should focus on pathway regulation by miRNAs rather than individual targets, in a cell or tissue context. Hence, new miRNA therapeutic tools could be designed in a tissue-directed approach.

It should be pointed out that although the other microRNAs found differentially expressed in the NGS step were not validated in the independent sample set, we cannot rule out that they could be involved in COVID-19 symptomatology, and patients MV+ had a particular circulating microRNA signature with more than one altered microRNA. Further analyses in a larger sample size should be addressed.

In this regard, a similar study (CIBERESUCICOVID) that included 84 participants identified a signature of three miRNAs (miR-148a-3p, miR-451a, and miR-486-5p) that distinguished between ICU patients and ward patients^38^. MiR-451a was also found to be altered in our discovery samples (Supplementary Table 2), suggesting a possible role of this miRNA in the severity of COVID-19. The different findings between both studies can be attributed to differences between the methodologies used to identify microRNAs (RT-PCR of candidate genes versus NGS of all circulating microRNAs).

In conclusion, miR-369-3p was found decreased in serum samples of COVID-19 patients with mechanical ventilation requirement compared to COVID-19 patients without this requirement. Along with in silico analyses and its previously described involvement in inflammation, this study adds new insights in the putative role of miR-369-3p in the immune response.

## Supplementary Information

Supplementary Information.

## References

[1] Boban M (2021). Novel coronavirus disease (COVID-19) update on epidemiology, pathogenicity, clinical course and treatments. Int. J. Clin. Pract..

[2] Cangiano B (2020). Mortality in an Italian nursing home during COVID-19 pandemic: Correlation with gender, age, ADL, vitamin D supplementation, and limitations of the diagnostic tests. Aging (Albany NY).

[3] Mehta P (2020). COVID-19: Consider cytokine storm syndromes and immunosuppression. Lancet.

[4] Diao B (2020). Reduction and functional exhaustion of T cells in patients with coronavirus disease 2019 (COVID-19). Front. Immunol..

[5] Jafarzadeh A, Chauhan P, Saha B, Jafarzadeh S, Nemati M (2020). Contribution of monocytes and macrophages to the local tissue inflammation and cytokine storm in COVID-19: Lessons from SARS and MERS, and potential therapeutic interventions. Life Sci..

[6] Bartel DP (2004). MicroRNAs: Genomics, biogenesis, mechanism, and function. Cell.

[7] Girardi E, Lopez P, Pfeffer S (2018). On the importance of host microRNAs during viral infection. Front. Genet..

[8] Tan L (2016). Recent advances of exosomes in immune modulation and autoimmune diseases. Autoimmunity.

[9] Lee HM, Kim TS, Jo EK (2016). MiR-146 and miR-125 in the regulation of innate immunity and inflammation. BMB Rep..

[10] O'Connell RM, Rao DS, Chaudhuri AA, Baltimore D (2010). Physiological and pathological roles for microRNAs in the immune system. Nat. Rev. Immunol..

[11] Tang H (2020). The noncoding and coding transcriptional landscape of the peripheral immune response in patients with COVID-19. Clin. Transl. Med..

[12] Dobin A (2013). STAR: ultrafast universal RNA-seq aligner. Bioinformatics.

[13] Liao Y, Smyth GK, Shi W (2014). featureCounts: An efficient general purpose program for assigning sequence reads to genomic features. Bioinformatics.

[14] Ritchie ME (2015). limma powers differential expression analyses for RNA-sequencing and microarray studies. Nucleic Acids Res..

[15] Robinson MD, Oshlack A (2010). A scaling normalization method for differential expression analysis of RNA-seq data. Genome Biol..

[16] Robinson MD, McCarthy DJ, Smyth GK (2010). edgeR: a Bioconductor package for differential expression analysis of digital gene expression data. Bioinformatics.

[17] Overmyer KA (2021). Large-scale multi-omic analysis of COVID-19 severity. Cell Syst..

[18] Yu G, Wang LG, Han Y, He QY (2012). clusterProfiler: An R package for comparing biological themes among gene clusters. OMICS.

[19] Subramanian A (2005). Gene set enrichment analysis: a knowledge-based approach for interpreting genome-wide expression profiles. Proc. Natl. Acad. Sci. USA.

[20] Liberzon A (2011). Molecular signatures database (MSigDB) 3.0. Bioinformatics.

[21] Hochberg Y, Benjamini Y (1990). More powerful procedures for multiple significance testing. Stat. Med..

[22] Trovato M (2021). Interleukin6 signalling as a valuable cornerstone for molecular medicine (Review). Int. J. Mol. Med..

[23] Matsuzaki J, Ochiya T (2018). Extracellular microRNAs and oxidative stress in liver injury: A systematic mini review. J. Clin. Biochem. Nutr..

[24] Wang SS (2018). A meta-analysis of dysregulated miRNAs in coronary heart disease. Life Sci..

[25] Shi C (2016). Adipogenic miRNA and meta-signature miRNAs involved in human adipocyte differentiation and obesity. Oncotarget.

[26] Xu Q (2021). LncRNA-ATB regulates epithelial-mesenchymal transition progression in pulmonary fibrosis via sponging miR-29b-2-5p and miR-34c-3p. J. Cell Mol. Med..

[27] Backes C, Meese E, Keller A (2016). Specific miRNA disease biomarkers in blood, serum and plasma: Challenges and prospects. Mol. Diagn. Ther..

[28] Cigna N (2012). The hedgehog system machinery controls transforming growth factor-beta-dependent myofibroblastic differentiation in humans: Involvement in idiopathic pulmonary fibrosis. Am. J. Pathol..

[29] Massague J, Weis-Garcia F (1996). Serine/threonine kinase receptors: Mediators of transforming growth factor beta family signals. Cancer Surv..

[30] Lenart M (2020). miRNA regulation of NK cells antiviral response in children with severe and/or recurrent herpes simplex virus infections. Front. Immunol..

[31] Wang J, Chen X, Huang W (2021). MicroRNA-369 attenuates hypoxia-induced cardiomyocyte apoptosis and inflammation via targeting TRPV3. Braz. J. Med. Biol. Res..

[32] Scalavino V (2020). miR-369-3p modulates inducible nitric oxide synthase and is involved in regulation of chronic inflammatory response. Sci. Rep..

[33] Jensen MD (2015). Circulating microRNAs as biomarkers of adult Crohn's disease. Eur. J. Gastroenterol. Hepatol..

[34] Yi H, Luo D, Xiao Y, Jiang D (2021). Knockdown of long noncoding RNA DLEU2 suppresses idiopathic pulmonary fibrosis by regulating the microRNA3693p/TRIM2 axis. Int. J. Mol. Med..

[35] Fulzele S (2020). COVID-19 virulence in aged patients might be impacted by the host cellular microRNAs abundance/profile. Aging Dis..

[36] Drury RE, O'Connor D, Pollard AJ (2017). The clinical application of microRNAs in infectious disease. Front. Immunol..

[37] Lu Q, Wu R, Zhao M, Garcia-Gomez A, Ballestar E (2019). miRNAs as therapeutic targets in inflammatory disease. Trends Pharmacol. Sci..

[38] Gonzalo-Calvo D (2021). Circulating microRNA profiles predict the severity of COVID-19 in hospitalized patients. Transl. Res..

