# Supplementary material for: Circulating microRNA profiling is altered in the acute respiratory distress syndrome related to SARS-CoV-2 infection
Source: Sci Rep. 2022 Apr 28;12:6929. doi: 10.1038/s41598-022-10738-3 (PMC9047579; doi:10.1038/s41598-022-10738-3)
Supplement: Supplementary file 1 — Supplementary Information. [file 41598_2022_10738_MOESM1_ESM.docx]

**Circulating microRNA profiling is altered in the acute respiratory distress syndrome related to SARS-CoV-2 infection**

Supplementary Material

**Medical Criteria for Mechanical Ventilation (MV) requirements:**

**MV+**

Admitted to the hospital by SARS-CoV-2 infection.

Severe Respiratory Failure defined as two of these three criteria: pO_2_ less than 60 mm Hg (or room air oxygen saturation less than or equal to 90%), pCO_2_ greater than 50 mm Hg with pH less than 7.35, and signs/symptoms of respiratory distress.

Requiring invasive mechanical ventilation to maintain oxygen saturation.

Admission to ICU.

**MV-**

Admitted to the hospital by SARS-CoV2 infection.

Respiratory Failure defined as two of these three criteria: pO_2_ less than 60 mm Hg (or room air oxygen saturation less than or equal to 90%), pCO_2_ greater than 50 mm Hg with pH less than 7.35, and signs/symptoms of respiratory distress.

Requiring oxygen supplementation to maintain Sat O2 >90% but without needing invasive mechanical ventilation nor non-invasive mechanical ventilation.

Admitted to a conventional hospitalization ward.

Supplementary Table 1: Characteristics and co-morbidities of patients included in the discovery sample.

**Abbreviations:** HBP, high blood pressure; CKD, Chronic kidney disease; DM, Diabetes Mellitus; CRD, Chronic respiratory diseases, CAD, cardiovascular disease.

Supplementary Table 2. Differentially expressed microRNAs between MV+ and MV- patients with a value of log-fold change larger than 1 and p-value below 0.05

| **miRNA ID** | **mean counts MV-** | | **mean counts MV+** | **FC MV+ vs MV-** | **LogFC MV+ vs MV-** | **P-Value** |
| --- | --- | --- | --- | --- | --- | --- |
| hsa-miR-142-3p | 14,04 | 12,82 | | -2,37 | -1,25 | 0,011 |
| hsa-miR-122-5p | 13,75 | 14,88 | | 2,30 | 1,20 | 0,030 |
| hsa-miR-26b-5p | 13,34 | 12,38 | | -2,09 | -1,06 | 0,020 |
| hsa-miR-142-5p | 12,63 | 11,52 | | -2,18 | -1,12 | 0,014 |
| hsa-miR-451a | 12,11 | 10,44 | | -2,60 | -1,38 | 0,012 |
| hsa-miR-101-3p | 11,62 | 9,96 | | -2,92 | -1,55 | 0,009 |
| hsa-miR-320b | 10,75 | 11,90 | | 2,59 | 1,37 | 0,008 |
| hsa-miR-19b-3p | 9,36 | 8,30 | | -2,03 | -1,02 | 0,025 |
| hsa-miR-1246 | 8,68 | 10,04 | | 3,08 | 1,62 | 0,006 |
| hsa-miR-182-5p | 8,54 | 7,14 | | -2,62 | -1,39 | 0,003 |
| hsa-miR-3182 | 8,20 | 9,95 | | 3,64 | 1,86 | 0,001 |
| hsa-miR-183-5p | 8,03 | 6,56 | | -2,49 | -1,32 | 0,018 |
| hsa-miR-326 | 7,82 | 6,98 | | -2,14 | -1,10 | 0,005 |
| hsa-miR-19a-3p | 7,68 | 6,38 | | -2,30 | -1,20 | 0,021 |
| hsa-miR-29b-3p | 7,61 | 6,23 | | -2,48 | -1,31 | 0,006 |
| hsa-miR-340-5p | 7,39 | 6,01 | | -2,85 | -1,51 | 0,005 |
| hsa-miR-431-5p | 7,31 | 6,46 | | -2,36 | -1,24 | 0,007 |
| hsa-miR-144-3p | 7,17 | 4,86 | | -3,87 | -1,95 | 0,012 |
| hsa-miR-574-3p | 6,88 | 8,25 | | 2,32 | 1,21 | 0,006 |
| hsa-miR-190a-5p | 6,79 | 5,50 | | -2,26 | -1,18 | 0,008 |
| hsa-miR-32-5p | 6,72 | 5,06 | | -2,88 | -1,53 | 0,022 |
| hsa-miR-374a-5p | 6,70 | 5,07 | | -3,56 | -1,83 | 0,005 |
| hsa-miR-381-3p | 6,55 | 5,90 | | -2,21 | -1,14 | 0,034 |
| hsa-miR-193a-5p | 6,46 | 8,34 | | 4,49 | 2,17 | 0,000 |
| hsa-miR-1277-5p | 6,37 | 4,62 | | -3,47 | -1,79 | 0,004 |
| hsa-miR-18a-5p | 6,34 | 5,39 | | -2,11 | -1,08 | 0,021 |
| hsa-miR-760 | 6,32 | 7,76 | | 2,96 | 1,57 | 0,004 |
| hsa-miR-144-5p | 6,09 | 4,14 | | -3,11 | -1,64 | 0,003 |
| hsa-miR-301a-3p | 5,91 | 4,50 | | -2,94 | -1,56 | 0,005 |
| hsa-miR-96-5p | 5,90 | 4,56 | | -2,39 | -1,25 | 0,013 |
| hsa-miR-2110 | 5,87 | 6,98 | | 2,22 | 1,15 | 0,012 |
| hsa-miR-30b-5p | 5,58 | 4,77 | | -2,06 | -1,05 | 0,027 |
| hsa-miR-1307-5p | 5,54 | 4,50 | | -2,17 | -1,11 | 0,031 |
| hsa-miR-15b-3p | 5,54 | 4,44 | | -2,40 | -1,26 | 0,012 |
| hsa-miR-576-5p | 5,48 | 3,96 | | -2,87 | -1,52 | 0,002 |
| hsa-miR-106b-5p | 5,33 | 3,73 | | -2,97 | -1,57 | 0,004 |
| hsa-miR-215-5p | 5,31 | 3,86 | | -2,15 | -1,10 | 0,042 |
| hsa-miR-374b-5p | 5,29 | 3,87 | | -3,21 | -1,68 | 0,007 |
| hsa-miR-374c-3p | 5,29 | 3,87 | | -3,21 | -1,68 | 0,007 |
| hsa-miR-590-3p | 5,10 | 3,63 | | -3,03 | -1,60 | 0,007 |
| hsa-miR-485-5p | 5,07 | 6,30 | | 2,13 | 1,09 | 0,031 |
| hsa-miR-628-5p | 5,00 | 3,60 | | -3,01 | -1,59 | 0,004 |
| hsa-miR-25-5p | 4,94 | 5,96 | | 2,10 | 1,07 | 0,017 |
| hsa-miR-4516 | 4,71 | 6,13 | | 3,88 | 1,95 | 0,003 |
| hsa-miR-548d-5p | 4,57 | 3,54 | | -2,00 | -1,00 | 0,036 |
| hsa-miR-3187-3p | 4,49 | 5,87 | | 2,83 | 1,50 | 0,001 |
| hsa-miR-16-2-3p | 4,47 | 3,18 | | -2,18 | -1,12 | 0,038 |
| hsa-miR-548ae-5p | 4,47 | 3,37 | | -2,03 | -1,02 | 0,041 |
| hsa-miR-548ap-3p | 4,45 | 3,30 | | -2,10 | -1,07 | 0,032 |
| hsa-miR-548ad-5p | 4,31 | 3,19 | | -2,06 | -1,04 | 0,037 |
| hsa-miR-181c-3p | 4,27 | 3,22 | | -2,51 | -1,33 | 0,002 |
| hsa-miR-33a-5p | 4,23 | 2,97 | | -2,76 | -1,47 | 0,007 |
| hsa-miR-92b-5p | 4,23 | 5,05 | | 2,29 | 1,19 | 0,027 |
| hsa-miR-335-3p | 4,22 | 3,29 | | -2,15 | -1,10 | 0,030 |
| hsa-miR-130b-5p | 4,17 | 3,37 | | -2,01 | -1,01 | 0,039 |
| hsa-miR-542-3p | 4,14 | 2,42 | | -2,87 | -1,52 | 0,009 |
| hsa-miR-874-3p | 4,12 | 4,99 | | 2,09 | 1,06 | 0,010 |
| hsa-miR-28-5p | 3,94 | 3,05 | | -2,09 | -1,06 | 0,043 |
| hsa-miR-320a | 3,92 | 5,31 | | 2,84 | 1,50 | 0,003 |
| hsa-miR-374a-3p | 3,88 | 2,04 | | -3,70 | -1,89 | 0,003 |
| hsa-miR-3679-5p | 3,87 | 5,31 | | 2,69 | 1,43 | 0,007 |
| hsa-miR-369-3p | 3,81 | 2,43 | | -3,44 | -1,78 | 0,005 |
| hsa-miR-148b-5p | 3,64 | 2,27 | | -2,62 | -1,39 | 0,006 |
| hsa-miR-1292-5p | 3,55 | 4,51 | | 2,09 | 1,06 | 0,016 |
| hsa-miR-342-5p | 3,51 | 4,93 | | 2,79 | 1,48 | 0,005 |
| hsa-miR-889-3p | 3,39 | 2,79 | | -2,18 | -1,12 | 0,035 |
| hsa-miR-23b-5p | 3,34 | 4,53 | | 2,07 | 1,05 | 0,017 |
| hsa-miR-885-3p | 3,29 | 4,58 | | 3,37 | 1,75 | 0,020 |
| hsa-miR-454-5p | 3,21 | 1,96 | | -2,70 | -1,43 | 0,009 |
| hsa-miR-146a-3p | 3,17 | 1,36 | | -3,39 | -1,76 | 0,012 |
| hsa-miR-23a-5p | 3,15 | 4,37 | | 2,27 | 1,19 | 0,014 |
| hsa-miR-193b-5p | 3,15 | 5,60 | | 6,28 | 2,65 | 0,002 |
| hsa-miR-1273h-5p | 3,12 | 4,74 | | 2,90 | 1,54 | 0,003 |
| hsa-miR-4429 | 3,05 | 4,03 | | 2,59 | 1,37 | 0,038 |
| hsa-miR-206 | 2,86 | 3,77 | | 2,57 | 1,36 | 0,026 |
| hsa-miR-6741-3p | 2,82 | 4,00 | | 2,18 | 1,12 | 0,020 |
| hsa-miR-136-3p | 2,74 | 1,72 | | -2,46 | -1,30 | 0,021 |
| hsa-miR-125a-3p | 2,73 | 4,40 | | 3,09 | 1,63 | 0,000 |
| hsa-miR-30d-3p | 2,71 | 1,41 | | -2,58 | -1,36 | 0,015 |
| hsa-miR-1297 | 2,67 | 1,47 | | -2,20 | -1,14 | 0,016 |
| hsa-miR-6741-5p | 2,64 | 3,82 | | 2,66 | 1,41 | 0,007 |
| hsa-miR-651-5p | 2,63 | 1,48 | | -2,22 | -1,15 | 0,040 |
| hsa-miR-181c-5p | 2,57 | 0,95 | | -3,60 | -1,85 | 0,002 |
| hsa-miR-450b-5p | 2,49 | 0,81 | | -3,02 | -1,59 | 0,007 |
| hsa-miR-376b-3p | 2,30 | 0,89 | | -3,10 | -1,63 | 0,006 |
| hsa-miR-6877-5p | 2,28 | 3,28 | | 2,10 | 1,07 | 0,012 |
| hsa-miR-4488 | 2,27 | 3,19 | | 2,90 | 1,54 | 0,029 |
| hsa-miR-377-3p | 2,12 | 0,99 | | -2,48 | -1,31 | 0,018 |
| hsa-miR-20a-3p | 2,11 | 0,32 | | -3,04 | -1,60 | 0,003 |
| hsa-miR-3127-5p | 2,09 | 3,57 | | 3,03 | 1,60 | 0,002 |
| hsa-miR-4284 | 2,09 | 3,78 | | 2,89 | 1,53 | 0,004 |
| hsa-miR-1303 | 2,02 | 2,96 | | 2,10 | 1,07 | 0,033 |
| hsa-miR-4710 | 2,01 | 3,56 | | 3,00 | 1,59 | 0,006 |
| hsa-miR-590-5p | 2,00 | 0,47 | | -2,91 | -1,54 | 0,018 |
| hsa-miR-548e-3p | 1,98 | 0,63 | | -2,48 | -1,31 | 0,021 |
| hsa-miR-1179 | 1,97 | 0,74 | | -2,34 | -1,23 | 0,017 |
| hsa-miR-205-5p | 1,96 | 3,05 | | 2,80 | 1,48 | 0,039 |
| hsa-miR-3198 | 1,92 | 2,91 | | 2,02 | 1,02 | 0,035 |
| hsa-miR-16-1-3p | 1,88 | 0,20 | | -3,28 | -1,72 | 0,010 |
| hsa-miR-7151-3p | 1,84 | 0,66 | | -2,60 | -1,38 | 0,007 |
| hsa-miR-301b-3p | 1,83 | 0,17 | | -3,19 | -1,68 | 0,003 |
| hsa-miR-8485 | 1,80 | 3,43 | | 3,02 | 1,60 | 0,009 |
| hsa-miR-1343-3p | 1,77 | 2,93 | | 2,29 | 1,19 | 0,039 |
| hsa-miR-374b-3p | 1,74 | 0,73 | | -2,43 | -1,28 | 0,028 |
| hsa-miR-1299 | 1,72 | 2,83 | | 2,71 | 1,44 | 0,039 |
| hsa-miR-150-3p | 1,68 | 3,42 | | 3,55 | 1,83 | 0,006 |
| hsa-miR-5010-3p | 1,67 | 0,44 | | -2,29 | -1,19 | 0,014 |
| hsa-miR-4738-3p | 1,67 | 2,97 | | 2,61 | 1,39 | 0,002 |
| hsa-miR-5698 | 1,61 | 2,82 | | 2,33 | 1,22 | 0,007 |
| hsa-miR-7704 | 1,56 | 2,76 | | 2,90 | 1,54 | 0,010 |
| hsa-miR-299-3p | 1,49 | 0,70 | | -2,25 | -1,17 | 0,045 |
| hsa-miR-504-5p | 1,49 | 2,65 | | 2,43 | 1,28 | 0,010 |
| hsa-miR-6891-5p | 1,43 | 2,41 | | 2,23 | 1,16 | 0,045 |
| hsa-miR-4485-3p | 1,31 | 2,28 | | 2,17 | 1,12 | 0,018 |
| hsa-miR-18b-5p | 1,28 | 0,04 | | -2,05 | -1,03 | 0,044 |
| hsa-miR-10b-3p | 1,23 | 2,23 | | 2,05 | 1,03 | 0,021 |
| hsa-let-7f-1-3p | 1,23 | 0,11 | | -2,52 | -1,33 | 0,022 |
| hsa-miR-619-5p | 1,22 | 2,11 | | 2,17 | 1,12 | 0,047 |
| hsa-miR-337-3p | 1,15 | 0,22 | | -2,03 | -1,02 | 0,038 |
| hsa-miR-2355-5p | 1,07 | -0,40 | | -2,65 | -1,41 | 0,022 |
| hsa-miR-4659a-5p | 1,07 | -0,05 | | -2,07 | -1,05 | 0,035 |
| hsa-miR-4748 | 1,07 | 2,09 | | 2,52 | 1,33 | 0,010 |
| hsa-miR-30b-3p | 1,04 | 2,28 | | 2,12 | 1,08 | 0,019 |
| hsa-miR-4504 | 1,03 | -0,53 | | -2,64 | -1,40 | 0,007 |
| hsa-let-7f-2-3p | 1,02 | -0,24 | | -2,67 | -1,42 | 0,016 |
| hsa-miR-545-3p | 0,96 | -1,04 | | -2,71 | -1,44 | 0,021 |
| hsa-miR-556-3p | 0,88 | -0,73 | | -3,35 | -1,75 | 0,008 |
| hsa-miR-6850-5p | 0,88 | 1,62 | | 2,35 | 1,23 | 0,029 |
| hsa-miR-556-5p | 0,78 | -0,73 | | -2,55 | -1,35 | 0,029 |
| hsa-miR-548ak | 0,78 | -1,04 | | -2,76 | -1,46 | 0,027 |
| hsa-miR-15a-3p | 0,77 | -1,22 | | -3,84 | -1,94 | 0,003 |
| hsa-miR-496 | 0,76 | -0,20 | | -2,50 | -1,32 | 0,011 |
| hsa-miR-624-5p | 0,76 | -0,70 | | -2,54 | -1,34 | 0,022 |
| hsa-miR-106a-3p | 0,75 | -0,81 | | -2,27 | -1,18 | 0,028 |
| hsa-miR-6783-5p | 0,72 | -0,46 | | -2,25 | -1,17 | 0,013 |
| hsa-miR-3180 | 0,61 | 1,55 | | 2,28 | 1,19 | 0,017 |
| hsa-miR-548o-3p | 0,59 | -0,54 | | -2,11 | -1,08 | 0,050 |
| hsa-miR-3154 | 0,59 | 1,69 | | 2,51 | 1,33 | 0,024 |
| hsa-miR-1228-5p | 0,58 | 1,50 | | 2,98 | 1,58 | 0,016 |
| hsa-miR-3180-3p | 0,58 | 1,42 | | 2,19 | 1,13 | 0,025 |
| hsa-miR-450a-2-3p | 0,57 | -0,44 | | -2,19 | -1,13 | 0,035 |
| hsa-miR-548f-5p | 0,56 | -1,09 | | -2,71 | -1,44 | 0,039 |
| hsa-miR-3115 | 0,54 | -0,46 | | -2,18 | -1,13 | 0,036 |
| hsa-miR-545-5p | 0,53 | -0,82 | | -2,56 | -1,36 | 0,046 |
| hsa-miR-548j-3p | 0,46 | -1,24 | | -2,59 | -1,37 | 0,019 |
| hsa-miR-1185-5p | 0,41 | -0,80 | | -2,11 | -1,08 | 0,031 |
| hsa-miR-376b-5p | 0,40 | -0,97 | | -2,54 | -1,35 | 0,009 |
| hsa-miR-376c-5p | 0,40 | -0,94 | | -2,55 | -1,35 | 0,009 |
| hsa-miR-642a-5p | 0,20 | 1,29 | | 2,08 | 1,05 | 0,027 |
| hsa-miR-642b-3p | 0,20 | 1,29 | | 2,08 | 1,05 | 0,027 |
| hsa-miR-374c-5p | 0,19 | -1,08 | | -2,91 | -1,54 | 0,008 |
| hsa-miR-6802-5p | 0,14 | 0,85 | | 2,16 | 1,11 | 0,034 |
| hsa-miR-1537-5p | 0,12 | -1,19 | | -2,31 | -1,21 | 0,043 |
| hsa-miR-6875-5p | 0,11 | 0,85 | | 2,10 | 1,07 | 0,048 |
| hsa-miR-1247-5p | -0,03 | 1,01 | | 2,91 | 1,54 | 0,027 |
| hsa-miR-4669 | -0,12 | 2,30 | | 5,70 | 2,51 | 0,009 |
| hsa-miR-3620-5p | -0,26 | -1,72 | | -2,51 | -1,33 | 0,045 |
| hsa-miR-135a-5p | -0,28 | -1,64 | | -2,45 | -1,29 | 0,030 |
| hsa-miR-3117-3p | -0,31 | -1,59 | | -2,51 | -1,33 | 0,037 |
| hsa-miR-3143 | -0,31 | -1,22 | | -2,32 | -1,21 | 0,034 |
| hsa-miR-1228-3p | -0,33 | 1,13 | | 3,82 | 1,93 | 0,008 |
| hsa-miR-548d-3p | -0,41 | -1,90 | | -2,36 | -1,24 | 0,042 |
| hsa-miR-1278 | -0,58 | -1,71 | | -2,53 | -1,34 | 0,024 |
| hsa-miR-6806-3p | -0,61 | -1,90 | | -2,19 | -1,13 | 0,043 |
| hsa-miR-4697-3p | -0,65 | 0,76 | | 3,88 | 1,96 | 0,048 |
| hsa-miR-6889-5p | -0,70 | 0,54 | | 2,64 | 1,40 | 0,021 |
| hsa-miR-3911 | -0,80 | -0,01 | | 2,15 | 1,10 | 0,047 |
| hsa-miR-937-5p | -0,98 | 0,03 | | 2,91 | 1,54 | 0,039 |
| hsa-miR-6796-5p | -1,07 | -0,16 | | 2,83 | 1,50 | 0,029 |
| hsa-miR-34c-3p | -1,52 | -0,43 | | 3,23 | 1,69 | 0,018 |

Supplementary Table 3. Validation results of 20 miRNAs using qPCR in an independent sample set of MV- vs. MV+ patients.

| miRNA ID | Average dcq MV- | Average dcq MV+ | SD MV- | SD MV+ | Fold change MV-/MV+ | t-test p-value |
| --- | --- | --- | --- | --- | --- | --- |
| hsa-miR-369-3p | -4.91 | -6.35 | 0.95 | 0.90 | 2.71 | 0.006 |
| hsa-miR-4516 | -3.15 | -2.06 | 1.38 | 1.26 | -2.12 | 0.082 |
| hsa-miR-320a | 3.95 | 3.58 | 0.58 | 0.26 | 1.29 | 0.093 |
| hsa-miR-144-3p | 0.75 | 1.52 | 1.20 | 1.03 | -1.70 | 0.144 |
| hsa-miR-125a-3p | -6.71 | -5.31 | 1.66 | 1.36 | -2.64 | 0.155 |
| hsa-miR-15a-3p | -6.44 | -5.86 | 0.60 | 0.59 | -1.49 | 0.179 |
| hsa-miR-16-1-3p | -6.88 | -6.11 | 1.13 | 0.55 | -1.70 | 0.185 |
| hsa-miR-193a-5p | -1.86 | -2.31 | 1.13 | 0.69 | 1.36 | 0.299 |
| hsa-miR-374a-5p | -0.97 | -0.68 | 0.76 | 0.80 | -1.22 | 0.419 |
| hsa-miR-181c-5p | -6.12 | -6.45 | 1.40 | 0.81 | 1.25 | 0.603 |
| hsa-miR-374a-3p | -6.64 | -6.74 | 1.55 | 0.95 | 1.07 | 0.905 |
| hsa-miR-502-3p | -3.04 | -3.02 | 0.50 | 0.23 | -1.01 | 0.928 |
| hsa-miR-23a-3p | 3.04 | 3.02 | 0.50 | 0.23 | 1.01 | 0.928 |
| hsa-miR-146a-3p | ND | ND | ND | ND | ND | ND |
| hsa-miR-150-3p | ND | ND | ND | ND | ND | ND |
| hsa-miR-206 | ND | ND | ND | ND | ND | ND |
| hsa-miR-34c-3p | ND | ND | ND | ND | ND | ND |
| hsa-miR-4488 | ND | ND | ND | ND | ND | ND |
| hsa-miR-556-3p | ND | ND | ND | ND | ND | ND |
| hsa-miR-885-3p | ND | ND | ND | ND | ND | ND |

**Abbreviations:** ND, non-detected in enough samples; SD: Standard Deviation

| Custom normalization with miR-502-3p and miR-23a-3p using the average of the assays detected in all samples (n=20 samples). |
| --- |
| A higher value indicates that the miRNA is more abundant in the particular sample. |

Supplementary Table 4. Integration of miR-369-3p targets with gene expression results from GSE157103. Significantly upregulated targets are shown.

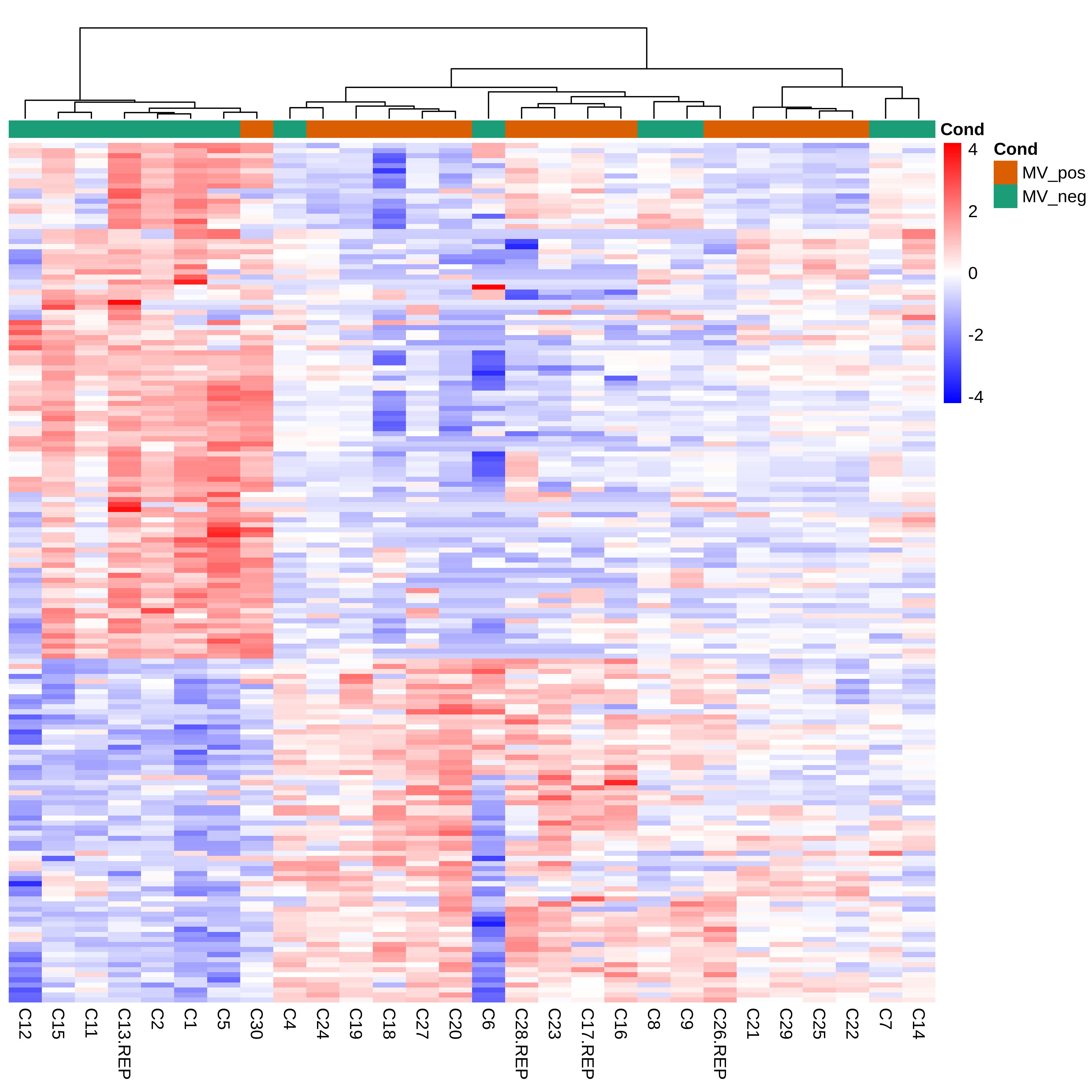


Supplementary Figure 1. Heatmap diagram of MV+ vs. MV- adjusted by SumRiskFact. The clustering is done using the complete-linkage method together with the Euclidean distance. Each row represents a microRNA and each column, a sample. The microRNA clustering tree is shown on the top. The colour scale illustrates the relative level of microRNA expression: blue, below the reference channel; red, higher than the reference.
